# Atomistic clustering-ordering and high-strain deformation of an Al_0.1_CrCoFeNi high-entropy alloy

**DOI:** 10.1038/srep31028

**Published:** 2016-08-08

**Authors:** Aayush Sharma, Prashant Singh, Duane D. Johnson, Peter K. Liaw, Ganesh Balasubramanian

**Affiliations:** 1 Mechanical Engineering, Iowa State University, Ames, IA 50011, USA; 2 Ames Laboratory, U.S. Department of Energy, Ames, IA 50011, USA; 3 Materials Science & Engineering, Iowa State University, Ames, IA 50011, USA; 4 Materials Science & Engineering, The University of Tennessee, Knoxville, TN 37996, USA; 5 Microelectronics Research Center, Iowa State University, Ames, IA 50011, USA

## Abstract

Computational investigations of structural, chemical, and deformation behavior in high-entropy alloys (HEAs), which possess notable mechanical strength, have been limited due to the absence of applicable force fields. To extend investigations, we propose a set of intermolecular potential parameters for a quinary Al-Cr-Co-Fe-Ni alloy, using the available ternary Embedded Atom Method and Lennard-Jones potential in classical molecular-dynamics simulations. The simulation results are validated by a comparison to first-principles Korringa-Kohn-Rostoker (KKR) - Coherent Potential Approximation (CPA) [KKR-CPA] calculations for the HEA structural properties (lattice constants and bulk moduli), relative stability, pair probabilities, and high-temperature short-range ordering. The simulation (MD)-derived properties are in quantitative agreement with KKR-CPA calculations (first-principles) and experiments. We study Al_x_CrCoFeNi for Al ranging from 0 ≤ x ≤2 mole fractions, and find that the HEA shows large chemical clustering over a wide temperature range for x < 0.5. At various temperatures high-strain compression promotes atomistic rearrangements in Al_0.1_CrCoFeNi, resulting in a *clustering-to-ordering* transition that is absent for tensile loading. Large fluctuations under stress, and at higher temperatures, are attributed to the thermo-plastic instability in Al_0.1_CrCoFeNi.

High-entropy alloys (HEAs) are solid solutions^1^,^2^,^3^,^4^,^5^,^6^,^7^,^8^ consisting of five or more metallic elements in approximately equimolar ratios with elemental compositions typically between 5–35 atomic percent (at.%). They have attracted increasing attention, especially as structural materials, due to their remarkable mechanical strength and resistance to oxidation and fatigue at ambient and elevated temperatures^3^,^4^,^5^,^9^,^10^. The compositional complexity does not automatically imply microstructural complexity due to the high mixing entropy. Often, an operational definition for HEAs is a solid-solution phase stabilized by the higher configurational entropy that increases with increasing temperature. With increasing the number of alloying elements (N), it should be noted, however, that the entropy increases as N *ln* N whereas interacting pairs that may drive chemical order increase as N^2^; so at low temperatures any favorable ordering enthalpy could overcome the slower increasing entropy – a point often not given its due importance. Thus, investigations of the HEA structural, chemical, and relative stability properties arising from the unique composition-structure relationship are alone intriguing, and deformation properties, in particular, have an important engineering role.

The deformation process in HEAs for quasi-static loading at low strain rates (10^−2^ to 10^−4^ s^−1^) reveals the contributions of slip and twinning mechanisms^11^,^12^,^13^,^14^,^15^. The deformation behavior at high-strain rates (<10^2^ s^−1^), even for conventional alloys undergoing dynamic loading, is complex due to the localized strain accumulating along the adiabatic shear bands^16^,^17^. High-strain rates in a crystalline lattice can lead to amorphization in the shear band that arise during deformation^18^, as shown by the formation of amorphous and nano-crystalline structures in the Fe-Ni-Cr alloy. Knowledge of high-strain deformation is particularly important for applications where strain rates higher than ~10^2^ s^−1^ are encountered^16^,^17^,^18^,^19^,^20^,^21^,^22^, such as high-temperature mechanical strength of lightweight armor materials, blast impact of debris on aircraft composite panels, satellites and spacecraft, as well as high-speed machining, all potentially relevant opportunities for employing HEAs. While research on HEAs is rapidly gaining interest, (1) the related literature on theoretical investigations is sparse and (2) experimental investigations are expensive and resource intensive. Additionally, because atomic arrangements in the alloy crystal significantly contribute to the material phase and deformation mechanics, we employ a combination of the computationally-demanding first-principles calculations and classical molecular-dynamics (MD) simulations to explore the high-strain deformation in a model HEA.

Classical MD simulations are unable to provide quantitatively accurate predictions of mechanical deformation for these multi-element alloys due to the lack of robust intermolecular potentials. Thus, earlier efforts in the literature have primarily resorted to computationally-expensive and system-size-limiting first-principles calculations, which are restricted in scope for understanding the deformation dynamics of very large systems. New potentials would enable a classical treatment of such alloys and allow us to examine much larger material domains consisting of several thousands of atoms.

Here, we derive relevant force-field parameters for a model quinary Al_0.1_CrCoFeNi HEA shown in Fig. 1, using the available ternary Embedded Atom Method (EAM) and Lennard-Jones (LJ) potentials. Structural properties (e.g., lattice constant, ‘*a*_0_’, and bulk modulus, ‘B’) are calculated for our model HEA using MD simulations; we then validate our force-field potentials by the comparison with available experiments and new first-principles Korringa-Kohn-Rostoker (KKR) - Coherent Potential Approximation (CPA) electronic-structure predictions. Besides structural properties, the KKR-CPA directly predicts the global and local relative stability of phases in HEAs, the Warren-Cowley short-range order (SRO) in the most stable, high-temperature solid-solution phase, as well as the underlying electronic origin for the chemical clustering or ordering behavior – pair by pair. Subsequently, our new force-field potential is utilized for calculating properties of Al_0.1_CrCoFeNi, in particular, the pair correlation function [g(|r**|**) or *g*_*α*−*β*_ (r)] that helps identify ordering and clustering mechanisms. The roles of temperature and quenching rates on the clustering-ordering characteristics in Al_0.1_CrCoFeNi are also investigated. Finally, the stress-strain analysis is performed under two different loading conditions (tension and compression) across a range of temperatures varying from cryogenic (77 K), room temperature (300 K), and at an elevated temperature (700 and 1,000 K). Our results show that clustering dominates over ordering tendencies in the Al_0.1_CrCoFeNi. However, high-strain compression can induce a *clustering-to-ordering* transition in these multi-element materials.

## Results and Discussion

The response of atoms to cluster or order in alloys offers deep insights to the microstructural behavior. This trend is particularly important for multi-component alloys, because the ordering can drive the system to various microstructural states, such as crystalline or intermetallic solids, from the disordered FCC (*A1*), BCC (*A2*), or HCP (*A3*) conditions. In Al_x_CrCoFeNi, the Al content can influence the final structural configuration^6^,^23^,^24^,^25^,^26^,^27^ due to the larger atom size, compared to other constituents, which considerably changes the lattice ordering. When the mole fraction of Al <0.3, a single-phase *A1* is observed, and a single-phase *A2* is found for Al >1.17, while a two-phase (*A1* + *A2*) for 0.3 ≤ Al ≤1.17 is reported^24^,^26^. Using the methods and approximations for accurate estimates of the formation enthalpy and relative energy of *A1* and *A2* phases^28^, the first-principles KKR-CPA results in Fig. 2 for Al of 0 < *x* < 2 mole fractions predict a similar global stability found in the experiment, i.e., *A1* for x < 0.5, *A1* + *A2* for 0.5 ≤ Al ≤1.25, and *A2* for Al > 1.25. While the *A1* phase is more stable than *A2* for smaller *x*, the positive formation enthalpies of *A1* and *A2* phases imply that clustering is expected in the HEA. Our findings are in agreement with experimental observations^24^ and semi-empirical CALPHAD calculations^29^, especially for x = 0.1. Hence, for subsequent simulations, we consider only the *A1* Al_x_CrCoFeNi with *x* = 0.10. In MD calculations, Al_0.1_CrCoFeNi is quenched from the melt phase, starting at 2,200 K. This arrangement ensures that the solid-solution alloy formed at 300 K is through the high-temperature melt phase at 2,200 K, as the experimental^24^ melting temperature reported for CrCoFeNi and Al_0.3_CrCoFeNi are around 1,690 K and 1,655 K, respectively.

We also calculate the Warren-Cowley short-range order (SRO) parameters, *α*_*αβ*_ (**k**), for all α-β pairs and for selected alloy compositions, with focus on Al_0.1_CrCoFeNi, using the thermodynamic linear-response based on the KKR-CPA method and its charge self-consistent potentials and densities. This fundamental approach identifies uniquely the chemical modes driving SRO through the chemical interchange energies, 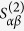 (**k**;T) – the thermodynamic cost to swap α and β atom types at two distinct sites, as reflected in the Fourier wave-vector, **k**. When 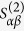
(**k**;T) > 0 and has a maximum at a particular, **k** = **k**_0_, for a specific α-β pair, it defines the unstable chemical mode and the dominant pair correlations (**k**_0_ = 0 indicates clustering, i.e., long wavelength “order”, and **k**_0_ ≠ 0 dictates finite-wavelength ordering with the **k**_0_ periodicity). While multiple pairs can have a peak at **k**_0_, a typical one will be dominant and other pairs will show correlations due to probability sum rules^28^. As shown in Fig. 3, the Al-Ni pair in 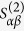
(**k**;T) possesses the strongest interchange-energy fluctuations at a long-wavelength given by **k** = [000] (a Γ-point for the *A1* lattice). These energies are manifested in the observable *α*_*αβ*_ (**k**) through the Al-Ni pair with a peak at **k** = [000] (i.e., Al-Al or Ni-Ni pairs clusters in real space), and a weak Al-Cr nearest-neighbor ordering at **k** = [001] (X-point). The diverging Al-Ni pair of *α*_*αβ*_ (**k = **[000]) at the spinodal temperature (T_sp_) indicates the absolute instability of the alloy that forces clustering in Al_0.1_CrCoFeNi, manifest in the Al-Al and Ni-Ni pairs, for example, and that can be compared to MD simulations from new force fields. The calculated spinodal temperature, T_sp_ = 840 K, shows good agreement with the experimentally-observed transition temperature of 813–823 K for Al_x_CrCoFeNi, 0 ≤ x ≤ 0.3^24^.

The variation of the cohesive energies (eV/atom) with the lattice constant (in Å) is calculated at 0 K, shown in Fig. 4, and compares first-principles and MD predictions with experiments. We predict the lattice parameter (*a*_0_) from KKR-CPA as 3.45 Å through local density approximation (LDA), 3.51 Å from the generalized gradient approximation (GGA), and 3.57 Å from the GGA corrected (GGA-c) for thermal expansion from zero-point phonon contributions from Grüneison theory, which is in agreement with room-temperature measurements (3.57 Å)^30^. The MD simulations find 3.55 Å at 0 K, in reasonable agreement with KKR and experiment. KKR-CPA also yield improved structural properties (e.g., lattice constant, ‘*a*_0_’, and bulk modulus, ‘B’) using GGA-c, i.e., *a*_0_ = 3.57 Å and B = 1.58 MPa at 300 K. MD simulations^31^,^32^ finds B by evaluating the curvature of the energy curve (Fig. 4) at *a*_0_ (3.55 Å). For the validation of the proposed EAM-LJ force-field, before analyzing structural and deformation behavior in Al_0.1_CrCoFeNi, a detailed quantitative comparison for ‘*a*_0_’ and ‘B’ for Al_x_CrCoFeNi at x = 0.1, 1.0 and 1.5 mole fraction are given in Table 1.

Structural pair correlation, g(r), in the real-space, as shown in Fig. 5[a–e], are derived from the MD simulations by histograms to identify (un)favorable neighboring atomic pairs constituting the alloy to predict the chemical mechanism (clustering or ordering) in Al_0.1_CrCoFeNi. We observe the strong affinity between the like pairs, i.e., Al-Al, Cr-Cr, Co-Co, Fe-Fe, and Ni-Ni. While the Al-Al pair correlation dominates all the like pairs, no significant contribution is noted from unlike pairs except Al-Cr [Fig. 5a]. The strong affinity of like pairs reveals clustering in Al_0.1_CrCoFeNi, in qualitatively good agreement with linear-response results (Figs 3 and 5). In Fig. 6, we illustrate pair probabilities calculated from the linear-response (first-principles) and force-field (MD) up to the 10^th^ shell in the real-space. The probability of finding unlike pairs at neighboring sites decreases with increasing the shell size. The difference in the Al-Ni probability is attributed to the fact that the “size effect”, which includes local site displacements is not included in the 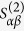
(**k**;T) calculations.

From MD simulations, the Al-Al pair correlations and trends at 500 K, 700 K, and 1,000 K in Fig. 7 are similar to the pair correlations found at 300 K. There is a marginal increase in the peak, g(r), of the Al-Al pairs in Fig. 7 while going from 300 to 500 K, which is attributed to an initial increase in the size of the Al-Al cluster size. However, as a temperature rise induces disorder in the atomistic rearrangement, this correlation decreases above 500 K in the simulated HEA.

The engineering stress versus engineering strain curves for Al_0.1_CrCoFeNi under dynamic compressive and tensile loadings at different temperatures from 77 K to 1,000 K are illustrated in Fig. 8[a,b], respectively. For compressive loading, a peak flow stress of ~125 MPa is recorded at 77 and 300 K, while it is ~90 MPa at 700 and 1,000 K. Above the flow stress, the thermo-plastic instability in the shear band causes thermal softening that dominates the stress-strain regime till an engineering strain of 0.6%. Thermal softening is more profound at elevated temperatures (700 K and above), as evident by the drastic drop in the flow stress at lower temperatures (77 K and 300 K). A consistent flow stress is observed until strain hardening. Strain hardening at various temperatures occurs at approximately similar strains, and beyond it the flow stress abruptly increases. The stress-strain curves for compressive loading show a marked evidence of strain hardening. The wide spectrum of thermo-plastic instability fluctuations at elevated temperatures is dissimilar to the instability found in shear bands of alloys under compressive loading conditions. In the case of tensile deformation [Fig. 8b], beyond the ultimate tensile stress (or critical flow stress) of ~75 MPa, a plastic flow occurs across the temperature range considered (77 K to 1,000 K). No strain hardening is observed under tensile deformation. Under compressive loading the clustering to ordering transition occurs with the increase in unlike pair correlations (e.g., Al-Co andAl-Cr) and decrease in like pair correlations (e.g., Al-Al), Fig. 9.

This prediction suggests that the quenched Al_0.1_CrCoFeNi evolves from a phase-separated to an ordered phase. However, for the corresponding tensile strain case, the clustering phase still dominates, as it induces a plastic flow condition, similar to a wire-drawing process. Thus, the transition to ordering state among the different elements of the Al_0.1_CrCoFeNi is possible only for the high compressive strain condition. At higher temperatures, we find large fluctuations in stress due to compressive loading. This thermo-plastic instability in Al_0.1_CrCoFeNi leads to shear-induced disordering similar to the structural changes arising in bulk metallic glasses (BMGs)^33^,^34^. Literature also suggests that mechanical alloying and shock compression of the Fe-Cu alloy system results in the *A1*-*A2* transformation^35^. Compression at the high-strain rate (10^10^ s^−1^) in our investigation is identical to an shock compression treatment^35^. The structural transformation of the *A2* Fe into *A3*/*A1* Fe phases under uniaxial compression has been observed in an earlier MD simulation^36^. Thus, the results of mechanical alloying and shock compression are essentially in accord with those from high-strain rate quenching and compression analysis, as presented here for Al_0.1_CrCoFeNi.

### Summary

In this report, we establish a force-field for a quinary Al-Cr-Co-Fe-Ni alloy and validate it by comparing structural properties, relative stability, and pair probabilities with first-principles calculations and limited experimental results. After the validation, we employ the new potential to investigate the large-scale deformation characteristics of Al_0.1_CrCoFeNi using atomistic MD simulations. Thus, we address the absence of well-developed force-field functions for high-entropy alloys and provide the EAM-LJ parameters to define the self- and cross-interactions of the participating elements in the alloy.

In Al_0.1_CrCoFeNi, the electronic-structure-based linear-response calculations predict clustering driven by Al-Ni pair correlations (forcing Al-Al and Ni-Ni clustering behavior). The MD simulations are in agreement with the clustering trend, as given by the large clustering domains of like pairs dominated by Al-Al. Although an increase in temperature reduces the clustering strength of like pairs, Al-Al correlations still dominate. The HEA shows a clustering-to-ordering transition under compressive loading, which is attributed to atomistic rearrangements at high strains. Corresponding pair correlations further corroborates the ordering behavior in the strained alloy. This investigation provides further motivation for the experimental exploration of high-temperature compressive thermo-plastic instability in Al_0.1_CrCoFeNi.

## Methods

### Electronic-structure

We investigate the phase stability of the solid-solution phase of the metallic Al_x_CrCoFeNi alloys (*x* = 0.0 – 2.0 mole-fraction) using the Korringa-Kohn-Rostoker (KKR) Green’s function method in combination with the screened Coherent Potential Approximation (CPA)^37^,^38^. The scalar-relativistic approximation is used for the valence electrons (no spin-orbit terms). The exchange-correlation functionals used are the von Barth-Hedin^39^ local density approximation (LDA) as parameterized by Moruzzi, Janak, and Williams^40^ and generalized gradient approximation (GGA) by Perdew-Burke-Ernzerhof revised for solids (PBEsol)^41^. A variational definition^42^ of the potential zero (v_0_) is utilized to yield the kinetic energies and dispersion that approach those of full-potential methods^42^,^43^. Potentials, charge densities, and total energies are obtained, using a complex-energy Gauss-Legendre semicircular contour with 24 points, and Brillioun-zone integrations use a special k-point method^44^ with a 20 × 20 × 20 mesh. Charge self-consistency is accelerated using the modified Broyden’s second method convergence technique^45^. Electronic properties and total energies are evaluated using the Voronoi polyhedra (VP) integration^46^ for spherically-averaged radial functions in the site-centered, spherical-harmonic (Y_L_) basis, where L = (*l, m*) is a composite angular quantum number referring to orbital (*l*) and spin (*m*). With L_max_ = 3, we include ***s-, p-, d-,*** and ***f**-*symmetries in the basis. To account for the thermal expansion at the finite temperature from phonons on the lattice constant and bulk modulus, we include the zero-point energy via the Grüneisen model^40^.

### Thermodynamic linear-response

The KKR-CPA grand potential (or free energy) is analytically expanded to second-order in compositional fluctuations for an arbitrary N-component alloy. The first-order terms vanish identically in a homogeneously-random (reference) state, whereas the second-order term gives the symmetric thermodynamic functional^47^ 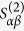
(**k**;T), quantifying chemical interchange energies and being analytically related to the atomic short-range order, SRO^47^,^48^. The KKR-CPA potentials, charge densities, and scattering matrices for a given solid solution are used to evaluate the linear-response expressions. 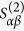
(**k**;T) includes all electronic structure, charge screening and transfer^47^, and it is no more costly to evaluate a binary alloy, as it is a HEA. Hence, the SRO (cluster or ordering) and its origin are related directly to the electronic structure, and provide insight into the competing effects, such as band-filling, atomic-size, Fermi-surface nesting, and charge-transfer. Notably, at a fixed composition, assuming that site charges vary little with SRO, Pettifor’s force theorem is applicable and, then only the band-energy variations for 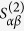
(**k**;T) survive^49^,^50^, and double-counting and exchange-correlation terms vanish^47^, simplifying the linear-response expressions. 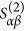
(**k**;T) is evaluated on a logarithmic frequency mesh containing all Matsubara poles, such that the response functions can be interpolated to the correct poles, i.e., temperature^51^,^52^,^53^. 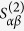
(**k**;T) is weakly temperature dependent from a Fermi factor, while 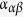
(**k**) strongly depends on temperature as the point entropy is analytically included, and as such it diverges at the spinodal temperature T_sp_ for a specific maximum wavevector^46^. 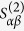
(**k**;T) is formulated in a “host” picture for the computational expediency. Then it is converted to an “off-diagonal” representation for the ease of comparison to the experiment^40^. The eigenvectors of the 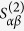
(**k**;T) chemical stability matrix just above T_sp_ reveal the ordering/clustering instability reflected in the SRO.

### Molecular-dynamics (MD) simulations

The highly-parallelized Large-scale Atomistic Molecular Massively Parallelized Simulator^54^ (LAMMPS) package is used for MD, while the visual analysis and post-processing of molecular trajectories is performed with Visual Molecular Dynamics^55^ (VMD). The MD simulations for investigating the structure of the Al_0.1_CrCoFeNi HEA^23^,^56^ have previously employed LJ potentials that cannot offer reliable predictions for such multi-component systems. Here, we assimilate the EAM/alloy potential parameters for Al, Co, Fe and Ni from the EAM database^57^,^58^ to model the elemental cross and self-interactions. Also, the cross interactions of Ni-Cr and Fe-Cr and the self-interactions of Cr-Cr are described with the EAM/alloy potential^57^. Only the Al-Cr and Co-Cr cross interactions are modeled, using the Lennard-Jones (LJ) potential^23^,^56^,^57^, due to the lack of the available EAM/alloy parameters for these interactions. The details of the functional forms of these atomic interactions are available in the literature^54^,^58^,^59^. We employ the Lennard-Jones potential (LJ) in the 12-6 form given by:


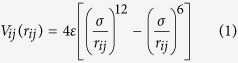


Here, *σ* is the distance where *V*_*ij*_(*σ*) = 0, and *ε* is the well depth of the LJ potential. The parameters for *σ* and *ε* considered in the present work are discussed in Table 2. These LJ parameters have been previously employed for different MD studies^56^,^60^. The Lorentz-Berthelot mixing rule is used to describe the cross interactions between Al-Cr and Co-Cr, such that for species, *i* and *j,* we have 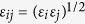 and 
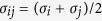
. We note that there is <2% deviation in the cohesive energy and negligible change in the system density with the variation in the cut-off radius for all the cross-interactions (Al-Cr and Cr-Co) described by the LJ-parameters in the present work. Thus, we employ a cut-off radius of r_cut-off_ = 10 Å in all our MD simulations to obtain accurate results in a reasonable amount of computational time.

**Figure 1:**
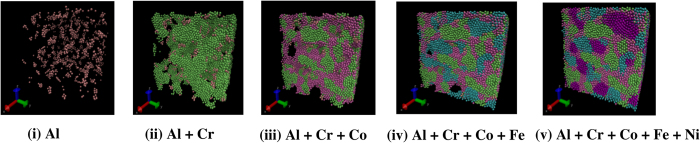
Atomistic representation of the high-entropy alloy for different elements on lattice. Elements (Al, Cr, Co, Fe, and Ni) are combined in a FCC lattice to form the Al_0.1_CrCoFeNi alloy. Quenching of Al_0.1_CrCoFeNi causes the high -T solid-solution disordered phase to shift to the phase-separated regions of (Al, Cr, Co, Fe, and Ni) at T = 300 K, as shown in (v).

**Figure 2:**
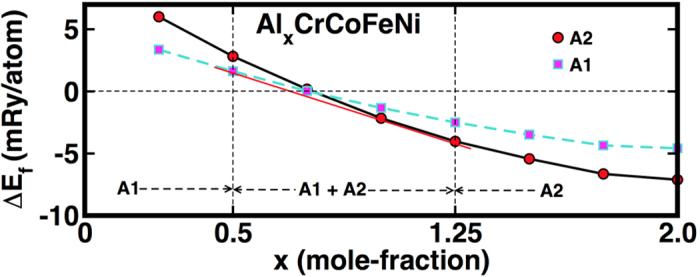
Phase stability of Al_x_CrCoFeNi (0 < x ≤2.0) from first-principles calculations. Phase stability of the *A1* (FCC) phase relative to *A2* (BCC) phase for Al_x_CrCoFeNi (x = 0.0–2.0, *x* in mole fraction) as calculated within the KKR-CPA-VP approach. The free energies of *A1* and *A2* phases are shown by solid-squares (magenta) and solid-circles (red), respectively. The formation of the *A1* phase is more favorable than *A2* phase for x ≤ 0.5. A common tangent line (red) to free-energy curves shows Al mole-fraction region (0.5 ≤ x ≤ 1.25) over which mixed phase (*A1* + *A2*) exists.

**Figure 3:**
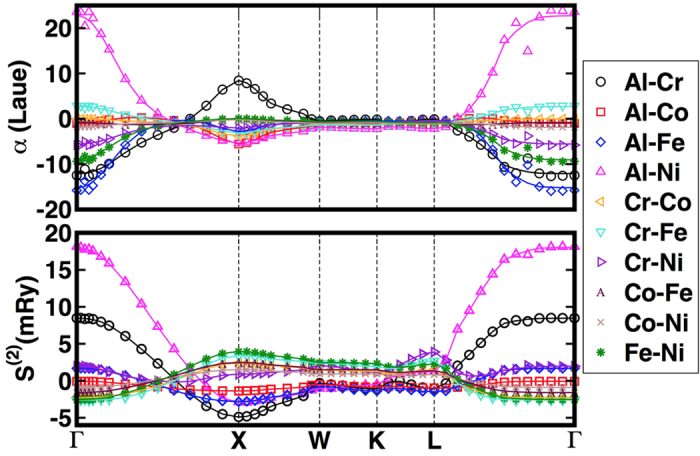
Short-range order and inter-change energies for Al_0.1_CrCoFeNi. Warren-Cowley short-range order [*α*_*αβ*_ (**k**), upper-panel] and chemical interchange-energies [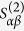 (**k**;T), lower panel] are presented along high-symmetry directions of the FCC Brillouin zone (Γ-X-W-K-L-Γ) for Al_0.1_CrCoFeNi. The clustering instability at Γ = (000) is driven by Al-Ni pairs in 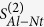 (**k** = Γ), and reflected in *α*_*αβ*_ (**k**).

**Figure 4:**
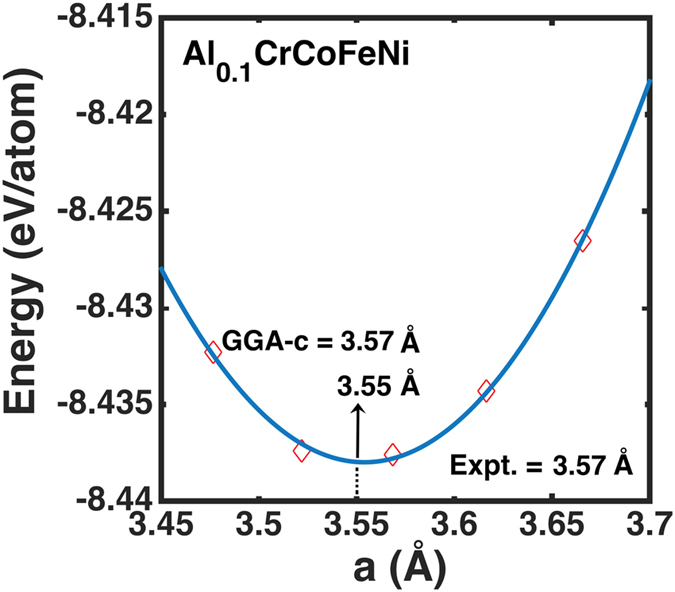
Energy vs. lattice parameter from MD simulations. Cohesive energies (diamonds in red) are shown as a function of lattice parameter calculated from MD simulations for Al_0.1_CrCoFeNi. Equilibrium lattice parameters from first-principles (3.57 Å from GGA-c, and 3.45 Å from LDA) and MD (3.55 Å) calculations are in good agreement with the experiment at room temperature (3.57 Å)^30^.

**Figure 5:**
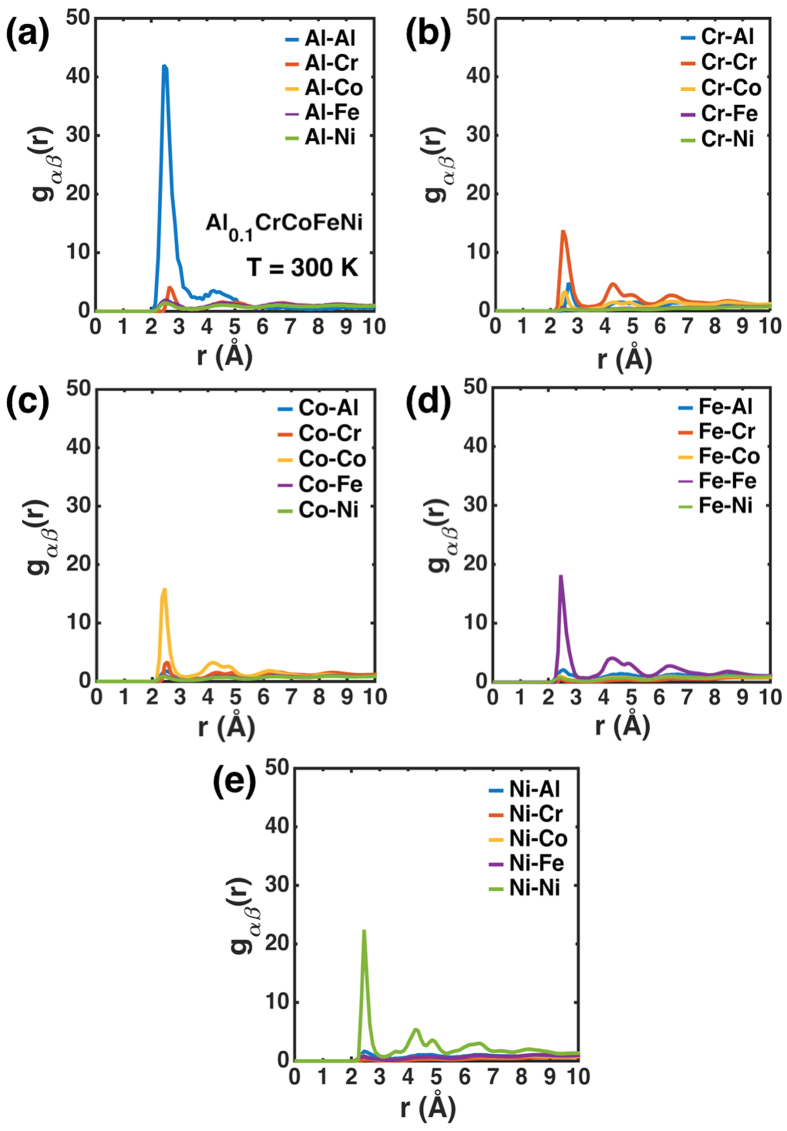
Pair correlation functions for Al_0.1_CrCoFeNi at 300 K. The pair correlations of like pairs (e.g., Al-Al) dominate over unlike pairs (e.g., Al-Cr) in the Al_0.1_CrCoFeNi at 300 K after a total simulation time (equilibration) of 4,000 ps, which indicates strong affinity among the like pairs for phase separation.

**Figure 6:**
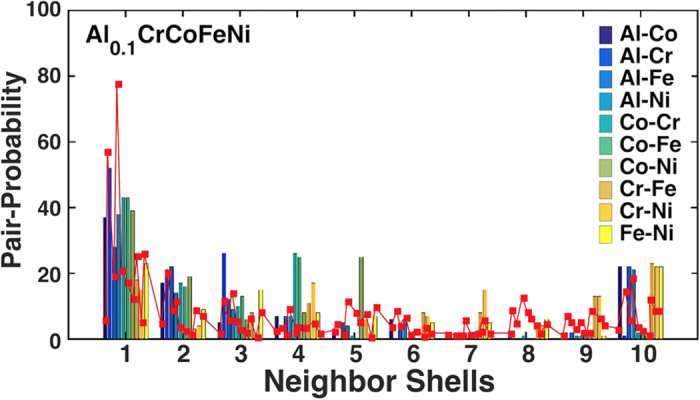
Real-space occupation probabilities. Pair probabilities in Al_0.1_CrCoFeNi up to 10^th^ shell at 300 K (quenched from 2,200 K) from the new force-field (histogram), which are compared with probabilities from the KKR-CPA linear response (red symbols) evaluated at 15% above the calculated spinodal (clustering) temperature of 840 K.

**Figure 7:**
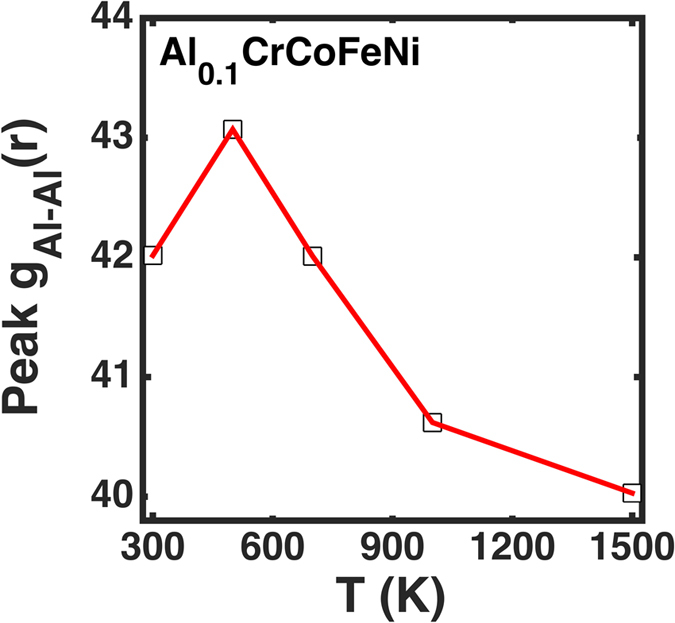
Absolute peak strength of Al-Al pair-correlations at varying temperatures in Al_0.1_CrCoFeNi. Peak g(r) increases initially from 300 K to 500 K, but drops above 500 K due to the reduced affinity among the like pairs.

**Figure 8:**
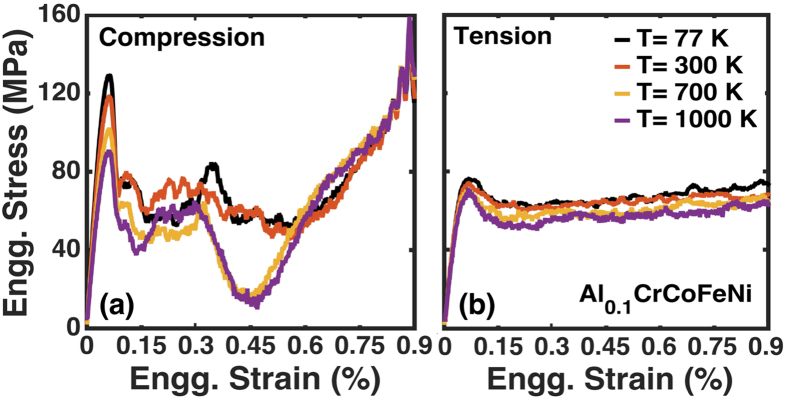
Engineering stress versus engineering strain for Al_0.1_CrCoFeNi at a strain rate of 10^10^ s^−1^. Stress under (**a**) compressive and (**b**) tensile load conditions at various temperatures – cryogenic (77 K), room temperature (300 K), and higher (700 K and 1,000 K). The tensile stress–strain diagram (**b**) shows only plastic deformation after the critical flow stress around 75 MPa for different temperatures, while the compressive stress-strain curve (**a**) shows the thermo-plastic instability leading to extended strain softening till a strain of 0.6%, followed by strain hardening.

**Figure 9:**
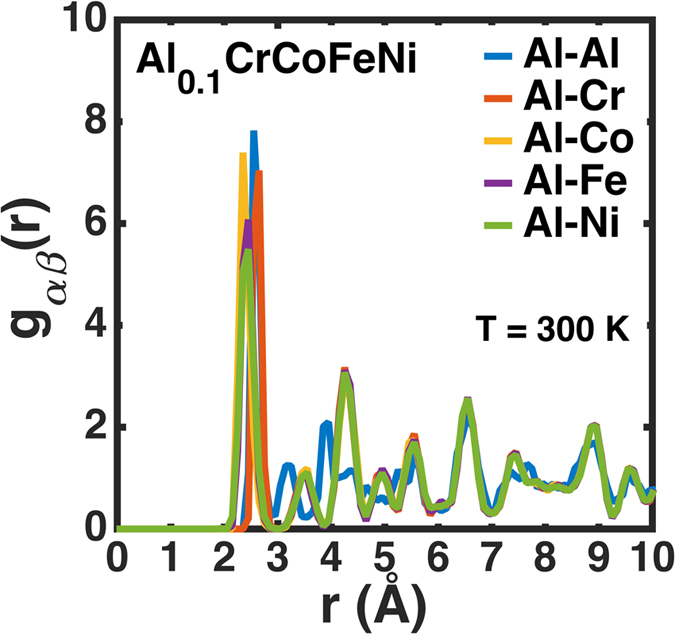
Pair correlation functions with uniaxial compression at a strain rate of 10^10^ s^−1^ for Al_0.1_CrCoFeNi at 300 K. Pair correlations of Al-Co, Al-Cr, and Al-Ni show the relatively strong tendency of ordering, which is a result of high strain deformation.

A FCC crystal lattice of 2.5 nm × 2.5 nm × 2.5 nm composed of randomly-distributed Al, Cr, Co, Fe and Ni atoms, shown in Fig. 1, is constructed with the elemental composition as described in Table 3. The simulation cell contains 62,500 atoms, and periodic boundary conditions are imposed in all directions. Energy minimization is carried out, using the steepest descent algorithm, with the energy and force tolerance set to 10^−15^ units (stopping tolerance for energy is unitless while force has a unit of eV/Å), to obtain the geometrically-optimized lattice configuration for the randomly-arranged Al_0.1_CrCoFeNi. The optimized structure is initialized at 2,200 K under an isothermal-isobaric (NPT) ensemble at a pressure of 0 MPa for 90 picoseconds (ps) to melt the alloy using equilibrium MD simulations. This step is followed by rapid quenching of the alloy under the NPT ensemble at 0 MPa with two different cooling rates of 21.11 K/ps and 5.42 K/ps, respectively, to reach the desired temperatures between 77 K and 1,500 K. We employ the Nosé-Hoover thermostat and barostat, each with a coupling time of 0.001 ps. Next, the structure is allowed to equilibrate for further 90 ps. A time step of 0.001 ps is maintained in all our MD simulations.

**Figure 10:**
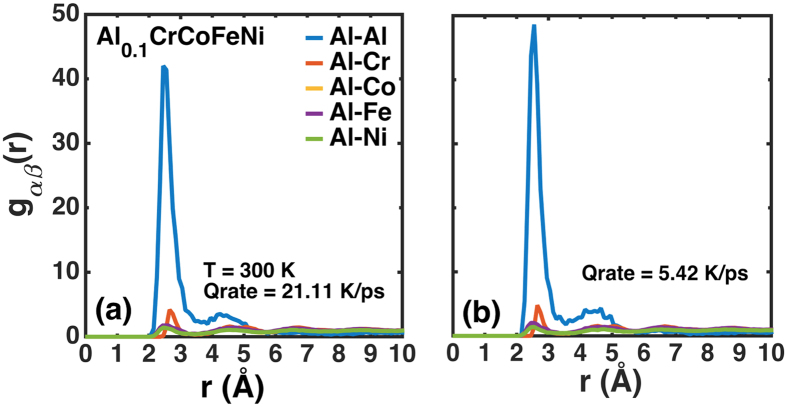
Effect of quenching rate on MD analysis of Al_0.1_CrCoFeNi. Pair correlations of Al with (Cr, Co, Fe, and Ni), as obtained at two different quenching rates (**a**) 21.11 K/ps and (**b**) 5.42 K/ps for Al_0.1_CrCoFeNi. No significant differences are predicted in the pair correlations for the different quenching rates. The pair correlations reveal strong tendencies for Al clustering.

As shown in Fig. 10[a,b], we observe no significant change in g(r) for different atomic pairs with varying quenching rates at a fixed temperature (300 K). Thus, for all simulations, in the present study, a quench rate of 21.11 K/ps is maintained. The quenched HEA is, then, further equilibrated under the NPT and NVT (canonical) ensembles successively. The pressure and temperature constraints each with the coupling time of 0.001 ps are imposed by the Nosé-Hoover thermostat and barostat, for a total time of 1 ns, followed by the NVT ensemble, for further 2 ns. Finally, the entire system is simulated in the absence of thermodynamic constraints for further 1 ns under the NVE (microcanonical) ensemble to ensure that we obtain an equilibrated structure. Next, tensile and compressive loadings of the alloy are performed independently at desired temperatures. The simulation cell is deformed in the *x*-direction of <100> with a strain rate of 10^10^ s^−1^, for the engineering strain of 0.9%, while lateral boundaries are controlled using the NPT equations of motion to zero pressure. Higher strain rates, ~10^10^ s^−1^, are chosen to provide predictions within reasonable computational wall-clock times due to length and time scale limitations in MD simulations. This trend restricts the use of experimentally-realizable high strain rates, ~10^2^ s^−1^. Nevertheless, deformation characteristics observed in our investigation are representative of those observed for experimentally-applied high strain rates. The atomic structures are analyzed from the molecular trajectories by pair correlations of different elements in the neighborhood of the chosen species.

## Additional Information

**How to cite this article**: Sharma, A. *et al.* Atomistic clustering-ordering and high strain deformation of Al_0.1_CrCoFeNi high-entropy alloy. *Sci. Rep.* **6**, 31028; doi: 10.1038/srep31028 (2016).

## Figures and Tables

**Table 1:** Structural properties of Al_x_CrCoFeNi, x = 0.1, 1.0 and 1.5 (or 2, 20, and 30% Al, respectively).

% Al	Method									
	Structure	KKR-CPA				MD				
		LDA		GGA-c		EAM-LJ			Expt.^24^,^30^	
		a_0_	B	a_0_	B	a_0_	B	*A2*-*A1*	a_0_	B
2	FCC	3.45	2.58	3.57	1.58	3.55	1.67	+11.84	3.57	1.50
20	FCC	3.51	2.08	3.63	1.77	3.70	1.84	−0.44	3.61	1.80
	BCC	2.80	2.31	2.83	2.21	3.00	3.70		2.88	—
30	BCC	2.82	1.55	2.86	1.57	2.95	2.10	−0.59	2.89	1.35

Comparing *a*_0_ (in Å) and B (in Mbar) from first-principles and classical MD simulations with the available experimental results. The GGA-c result includes zero-point energy correction from phonons via the Grüneisen model^40^. We also calculate the phase energy difference, *A2* - *A1* (in mRy), using the EAM-LJ potential, which shows the qualitative agreement with first-principles calculations (see Fig. 2).

**Table 2:** LJ potential parameters for describing Al, Cr, and Co interactions in our MD simulation of Al_0.1_CrCoFeNi.

Element	*ε* (eV)	σ(Å)
Al	0.392	2.620
Cr	0.502	2.336
Co	0.510	2.306

**Table 3:** Elemental compositions of different elements in Al_0.1_CrCoFeNi.

Percent	Element				
	Al	Cr	Co	Fe	Ni
Atomic	2	25	24	25	24
Mole	0.1	0.23	0.22	0.23	0.22
